# Jiedu Sangen Decoction Inhibits Migration and Invasion of Colon Cancer SW480 Cells via Suppressing Epithelial Mesenchymal Transition

**DOI:** 10.1155/2018/1495768

**Published:** 2018-09-25

**Authors:** Kai Zhang, Tao Peng, Qingying Yan, Leitao Sun, Haojun Miao, Li Yuan, Yuxin Hong, Mengmeng Zhou, Kaibo Guo, Leyin Zhang, Jing Fang, Minhe Shen, Shanming Ruan

**Affiliations:** ^1^The First Clinical Medical College of Zhejiang Chinese Medical University, Hangzhou 310053, China; ^2^Human Oncology and Pathogenesis Program, Memorial Sloan Kettering Cancer Center, New York 10065, USA; ^3^Department of Oncology, Shaoxing City Keqiao District Hospital of Traditional Chinese Medicine, Shaoxing 312030, China; ^4^Department of Traditional Chinese Internal Medicine, First People's Hospital of Yuhang District Hangzhou, Hangzhou 310000, China; ^5^Department of Medical Oncology, The First Affiliated Hospital of Zhejiang Chinese Medical University, Hangzhou 310006, China

## Abstract

Jiedu Sangen Decoction (JSD), a traditional Chinese medicine (TCM) formula, has been widely used in China to treat gastrointestinal cancer, especially as an adjuvant therapy in colorectal cancer (CRC) patients. This study aimed to evaluate the efficacy of JSD and Jiedu Sangen aqueous extract (JSAE) in colon cancer cells and explored the underlining mechanisms by cytotoxicity assay, scratch assay, transwell migration assay, matrigel invasion assay, confocal laser scanning microscopy, and western blot analysis. We demonstrated that JSAE inhibited the growth of colon cancer SW480 cells in a dose-dependent manner and JSAE repressed cancer cell migration and invasion. Furthermore, epithelial mesenchymal transition (EMT) was reversed by JSAE via enhancing E-cadherin expression and attenuating protein levels of EMT promoting factors such as N-cadherin, Slug, and ZEB1. These findings provided the first experimental evidence confirming the efficacy of JSAE in repressing invasion and metastasis of CRC and paving a way for the broader use of JSD in clinic.

## 1. Introduction

Colorectal cancer (CRC) is the third leading cause of cancer new cases in both males and females [1]. Studies have indicated that more than 80% of CRC cases and deaths are attributed to diet and environmental factors [2]. Most CRC patients have a very poor prognosis, with the 5-year survival rates dropping dramatically to 35% for those with lymph node involvement (stage III) and to 10% when the disease has spread to distant organs (stage IV) [3, 4]. Metastasis is the most decisive and lethal event during colon cancer course [5]. Therefore, preventing recurrence and metastasis is critical for colon cancer treatment.

Epithelial mesenchymal transition (EMT) is a crucial mechanism for CRC metastasis in the distant organ [6, 7]. EMT not only participates in normal embryonic development but also associates with various pathologic processes, including fibrosis and carcinogenesis. EMT is therefore clearly involved in cancer invasion and metastasis to distant tissues [8, 9]. E-cadherin is the characterized molecular marker of EMT, as loss of E-cadherin expression is known as the predominant hallmark of EMT [10, 11].

Jiedu Sangen Decoction (JSD), consisting of Teng Li Gen (Radix Actinidiae Argutae), Shui Yang Mei Gen (Root of Thinleaf AdinaI), and Hu Zhang Gen (Polygoni Cuspidati Radix), is an oral medicinal concoction that has been widely used to treat CRC patients in China. JSD decreased expression levels of TGF-*β* and MMP-9 secreted by carcinoma-associated fibroblasts and lowered mRNA levels of *α*-SMA and TGF-*β*, thereby inhibiting migration capability of colon cancer CT-26 cells [12]. However, the inhibitory mechanism of JSD remains elusive.

JSD has been proven to relieve symptoms and improve life quality of CRC patients in clinic. Based on our previous studies, we hypothesized that JSD may suppress EMT, resulting in repressing migration and invasion of CRC cells. In this report, we demonstrated that JSD suppressed the EMT and inhibited the migration and invasion of human colon cancer cells, by increasing E-cadherin expression that is an epithelial cell marker reciprocally decreasing N-cadherin expression that is a mesenchymal cell marker.

## 2. Material and Methods

### 2.1. Preparation of JSD and JSAE

Teng Li Gen (Radix Actinidiae Argutae): Shui Yang Mei Gen (Root of Thinleaf AdinaI) and Hu Zhang Gen (Polygoni Cuspidati Radix) were mixed equally and soaked with 8 times volume of distilled water for 30 min, before being simmered for 1 h after initial short hard boil. The first batch of decoction was collected, and the herbs were decocted again with 3 times volume of distilled water for another 25 min. All decoctions were merged and filtered, concentrated to 1.2g/ml as JSD, and stored at 4°C.

Two hundred grams of Teng Li Gen (Radix Actinidiae Argutae), Shui Yang Mei Gen (Root of Thinleaf AdinaI), and Hu Zhang Gen (Polygoni Cuspidati Radix) was pulverized to coarse powder, which was decocted with 8 and 6 times volume of distilled water, respectively, for 2 h. The aqueous extracts were merged and vacuum evaporated to recover the solvent to 2 g/ml as Jiedu Sangen aqueous extract (JSAE) and stored at 4°C.

### 2.2. Animals

Healthy male specific pathogen free Sprague-Dawley rats were purchased from experimental animal center of Zhejiang Chinese Medical University (production license number: SCXK (Shanghai) 2013-0016) at 120-200 g of weight, which were maintained at temperature ranging from 18 to 22°C and humidity of 60%-80% with free access to a standard diet and sterile water and a 12 h light/dark cycle. After one-week acclimation, 105 SD rats were randomly sorted into 3 treatment groups: JSD medicated serum (JMS) group (55 rats), normal saline serum (NSS) group (25 rats), and control serum (CS) group (25 rats). The rats in JMS group were treated orally with 1 ml/100g JSD b.i.d for four days. The rats in NSS group were treated orally with 1 ml/100g normal saline b.i.d for four days. The rats in CS group were raised normally for four days. All the rats were anesthetized with 3% pelltobarbitalum natricum at 0.15ml/100g. Blood samples from each rat were collected into 1.5 ml sterilized microtubes and kept on ice for 2 hours, which were then centrifuged at 4°C at 3000 RPM for 30 minutes. Supernatants were stored at −80°C for further research. All the animal experiments were approved by the Laboratory Animal Management and Welfare Ethical Review Committee of Zhejiang Chinese Medical University.

### 2.3. Cell Culture

The human colon cell line SW480 was purchased from cell resource center of the Institute of Basic Medical Sciences Chinese Academy of Medical Sciences and was cultured in Eagle's Minimum Essential Medium supplemented with 10% fetal bovine serum, 100 U/ml penicillin, and 100 mg/ml streptomycin. The cells were incubated in 5% CO_2_ at 37°C.

The cells were divided into seven groups: control, EGF, CS, NSS, JSAE, 10% JMS, and 15% JMS. Those in control group were cultured normally, and cells in the remaining six groups were treated with 50 ng/ml EGF for 48 h to induce EMT [13, 14]. After EMT was induced, the cells in JSAE group were cultured with 6 mg/ml JSAE for 48 h; those in CS group were cultured with 10% normally raised rats' serum for 48 h; those in NSS group were cultured with 10% normal saline treated rats' serum for 48 h; those in 10% JMS group were cultured with 10% JSD treated rats' serum for 48 h; those in 15% JMS group were cultured with 15% JSD treated rats' serum for 48 h.

### 2.4. Cytotoxicity Assay

Different concentrations of JSAE were assessed for their cytotoxic activities observed in SW480 cells. Briefly, the cells were incubated in a 96 well flat-bottomed microtiter plate. After being cultured for 12 h, the cells were treated with JSAE at a dose of 5, 10, 20 and 40 mg/ml in 200 ul media for 48 h. Cell viability was evaluated with Cell Counting Kit-8 reagent (Dojindo, Japan) according to the instructions of the manufacturer. The optical density values were measured at 450 nm using a microplate reader (Perkin-Elmer, USA). The results were analyzed by the linear regression probit model. These experiments were repeated three times.

### 2.5. Scratch Assay

The cells (1 × 10^5^) were seeded in a 6-wells plate in serum-free medium. A single stripe was scraped on the cell-coated surface with a 10 *μ*l plastic pipette tip. The cells were divided into seven groups as previous described. Migration was analyzed at 12 h using light microscopy (40X) with Image-Pro Plus Version 6.0. These experiments were repeated three times.

### 2.6. Transwell Migration Assay

The cells (2 × 10^4^) were plated into the top side of a polycarbonate transwell filter (8*μ*m, 24well; Corning, USA) and incubated at 37°C for 48 h. The cells inside the upper chamber were removed with cotton swabs. The cells on the lower membrane surface were fixed, stained with crystal violet, and counted for nine random ×400 fields per well. Cell counts are expressed as the mean number of cells per field of view. These experiments were repeated three times.

### 2.7. Matrigel Invasion Assay

Forty microliters of diluted matrigel was used to coat each of the upper chamber of transwell, and 2 × 10^4^ cells were seeded onto the upper chambers and cultured at 37°C for 48 h in a 5% CO_2_ incubator. The cells inside the upper chamber were then removed with cotton swabs. The cells on the lower membrane surface were fixed, stained with crystal violet, and counted for nine random ×400 fields per well. Cell counts were expressed as the mean number of cells per field of view. These experiments were repeated three times.

### 2.8. Confocal Laser Scanning Microscopy

Cell slide was fixed in 4% paraformaldehyde for 30 min and incubated with diluted primary antibodies (E-cadherin 1:100) at 4°C overnight. Afterwards, the cells were incubated with secondary antibodies (1:400) at room temperature in the dark for 50 min. With the addition of DAPI staining solution, the cells were further incubated at room temperature for 10 min in the dark. The coverslip was mounted on glass slide using antifade fluorescence mounting medium. The cells were observed at magnification ×400 under the confocal microscope and photographed. These experiments were repeated three times.

### 2.9. Western Blot Analysis

The cells were collected and lysed in RIPA buffer containing protease inhibitors. Protein concentration was determined with BCA assay kit, and equal amounts of protein (30 *μ*g) were electrophoresed on 10% sodium dodecyl sulfate-polyacrylamide gel and transferred onto polyvinylidene difluoride membranes (Bio-Rad, USA). Blots were blocked in 5% nonfat milk in TBST (TBS buffer containing 0.1% tween), incubated with the primary antibody at 1:1000 dilution overnight at 4°C, washed in TBST, and then incubated with secondary antibody at 1:5000 dilution for 2 h at room temperature. Signal was visualized by adding ECL detection reagent (Bio-Rad, USA). These experiments were repeated three times.

### 2.10. Statistical Analysis

All data are presented as the mean ± SD. The significance of the difference between groups was evaluated by one-way repeated-measures analysis of variance (ANOVA) with SPSS (version 22.0; SPSS, Inc., USA).* P *< 0.05 was considered to be statistically significant difference.

## 3. Results

### 3.1. JSAE Increases Cell Death in SW480 Human Colon Cancer Cells

**Figure 1:**
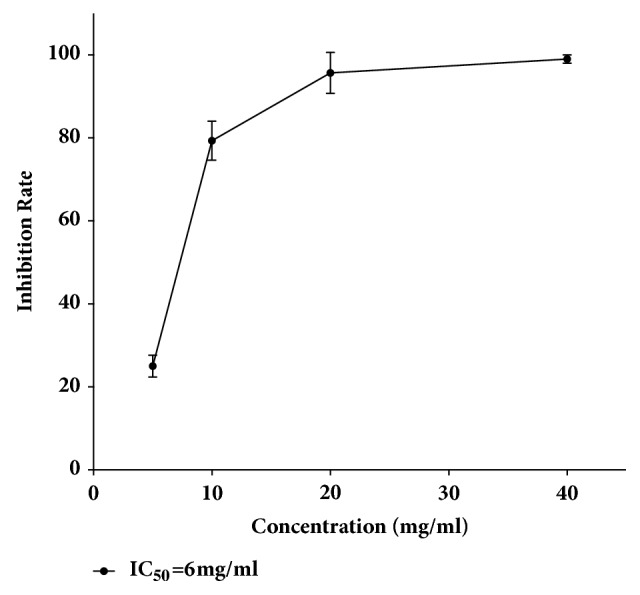
JSAE increases cell death of SW480 cells. Cells were treated with JSAE at the concentration of 5, 10, 20, and 40 mg/ml for 48 h. Cell viability was measured and linear regression analysis of probit model showed that IC_50_ of JASE was 6 mg/ml at 48 h.

To explore functional impact of JSAE on SW480 cells, cell viability assay was performed. Results showed that cytotoxicity of JSAE increased with concentrations; i.e., the higher the concentration, the greater the inhibitory effect. Linear regression analysis showed that the IC_50_ of JSAE was 6 mg/ml at 48 h (Figure 1).

### 3.2. JSAE Inhibited Migration and Invasion of SW480 Cells

**Figure 2:**
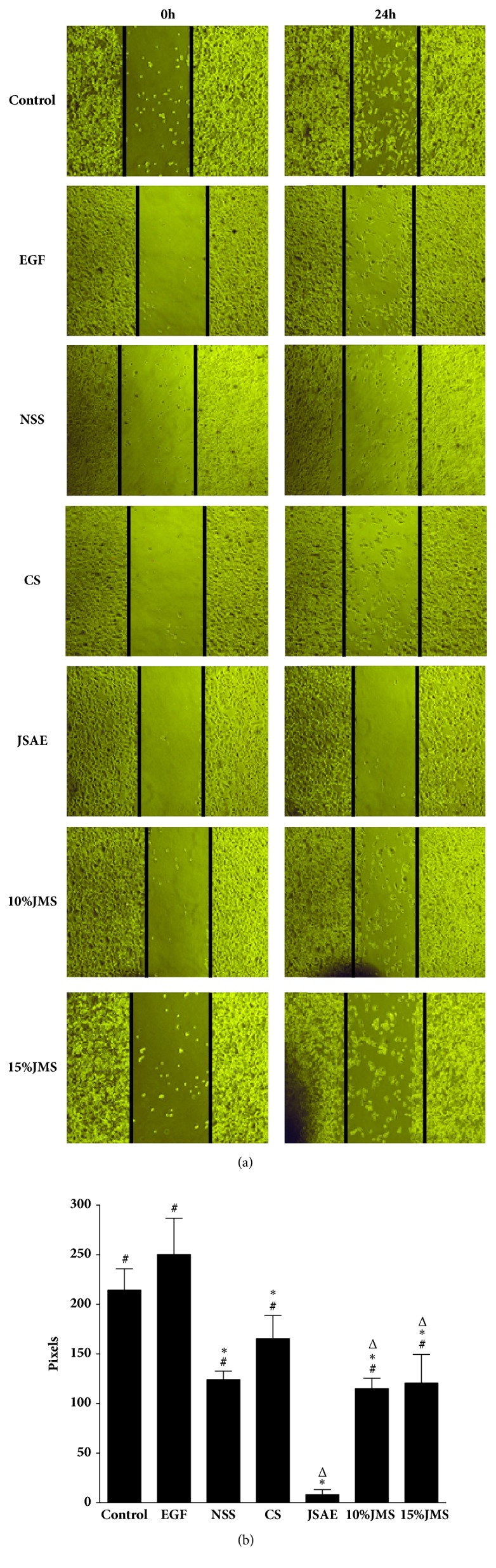
JSAE inhibits migration of SW480 cells demonstrated by scratch assay. SW480 cells treated with JSAE and different serum were subjected to scratch assay and observed at 0 and 24 h, respectively. (a) Representative images. (b) Quantifications of migration distance. ^*∗*^*P < *0.05, compared with EGF group; ^△^*P < *0.05, compared with control group; ^#^*P < *0.05, compared with JSAE group.

**Figure 3:**
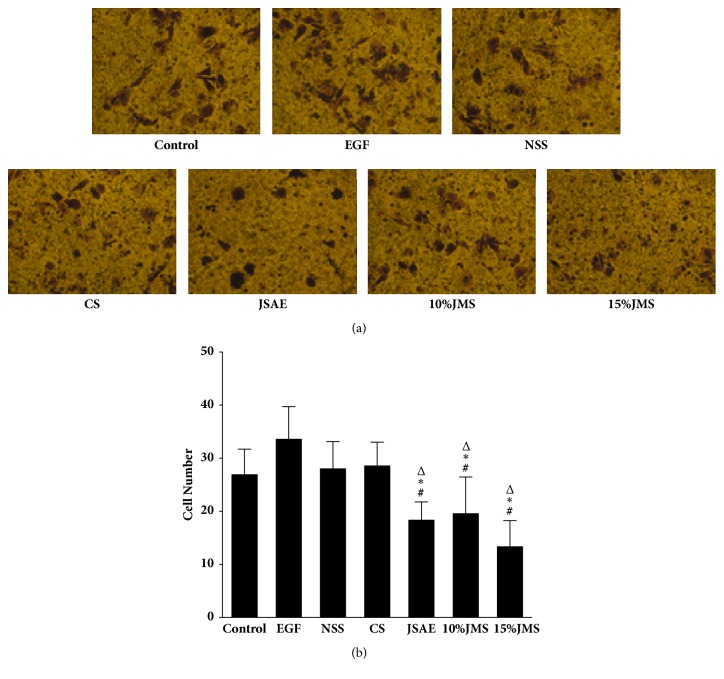
JSAE inhibits migration of SW480 cells demonstrated by transwell assay. SW480 cells treated with JSAE and different serum were subjected to transwell migration assay and observed at 48 h, respectively. (a) Representative transwell migration assay images. (b) Quantifications of cells penetrating the membrane. ^*∗*^*P* < 0.05, compared with EGF group; ^△^*P* < 0.05, compared with CS group; ^#^*P* < 0.05, compared with NSS group.

**Figure 4:**
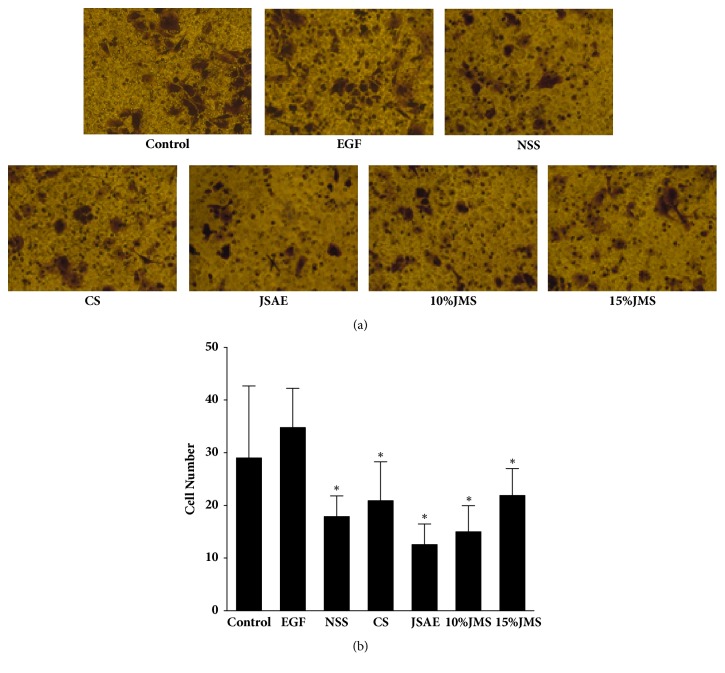
JSAE inhibits invasion of SW480 cells. SW480 cells treated with JSAE and different serum were subjected to matrigel invasion assay and observed at 48 h, respectively. (a) Representative matrigel invasion assay images. (b) Quantifications of cells penetrating the membrane. ^*∗*^*P* < 0.05, compared with EGF group.

JSAE decreased migration of SW480 cells in a scratch assay (Figure 2). In detail, at 24 h, JSAE < 10% JMS < 15% JMS < NSS < CS < control < EGF. Compared with EGF group, the migratory distance of JSAE group decreased significantly. Furthermore, we found that number of migratory cells decreased in transwell migration assay for both JSAE and JMS groups (Figure 3): 15% JMS < JSAE < 10% JMS < control < NSS < CS < EGF. The numbers of migratory cells in 10% and 15% JMS groups decreased relative to NSS and CS groups. Compared with EGF group, the JSAE group had a lower number of migratory cells. Using matrigel invasion assay we found that JSAE reduced the number of invasive cells (Figure 4): JSAE < 10% JMS < NSS < CS < 15% JMS < control < EGF. JSAE group showed an obvious reduction of number of cells penetrating the matrigel layer compared with EGF group.

### 3.3. JSAE Inhibited the EMT of SW480 Cells

**Figure 5:**
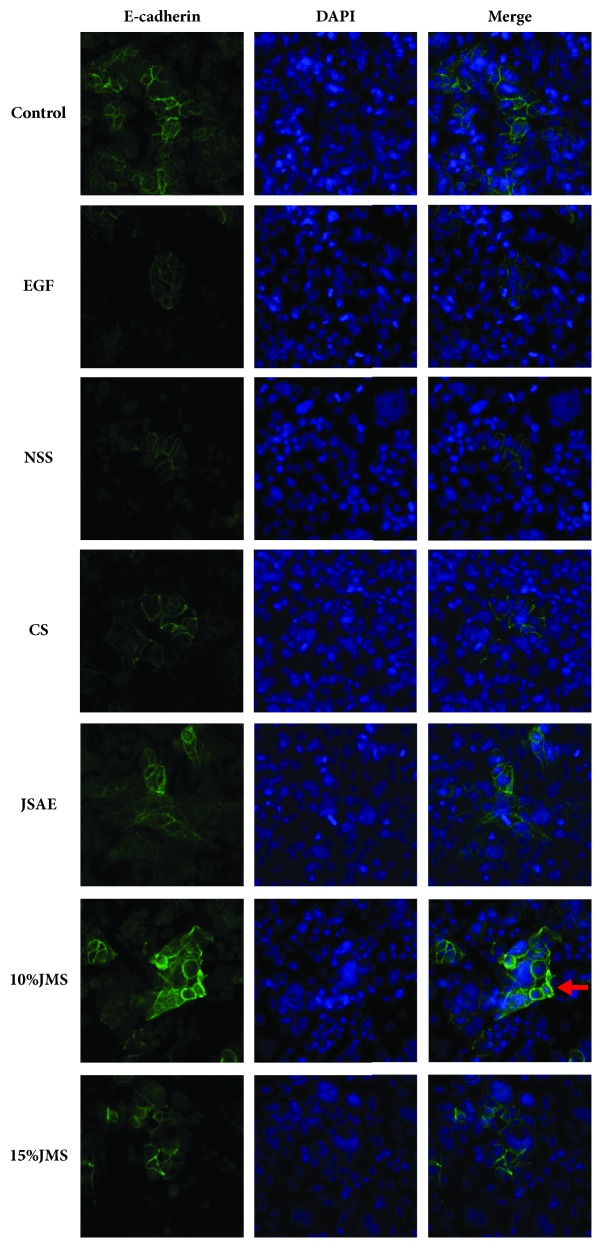
JSAE inhibits EMT of SW480 via upregulating E-cadherin expression after 48 h. Compared with control group, E-cadherin was downregulated after treating with EGF and 10% JMS significantly enhanced E-cadherin, indicated by red arrows, as well as JSAE.

E-cadherin, a key protein to EMT process, localizes in the cell membrane and the mesenchymal space [15, 16]. Through confocal laser scanning microscopy (Figure 5), we found that E-cadherin protein expression was upregulated in 10% and 15% JMS groups, as well as in JSAE group compared with NSS, CS, and EGF groups, respectively. This result suggested JSAE inhibited EMT by upregulating E-cadherin.

**Figure 6:**
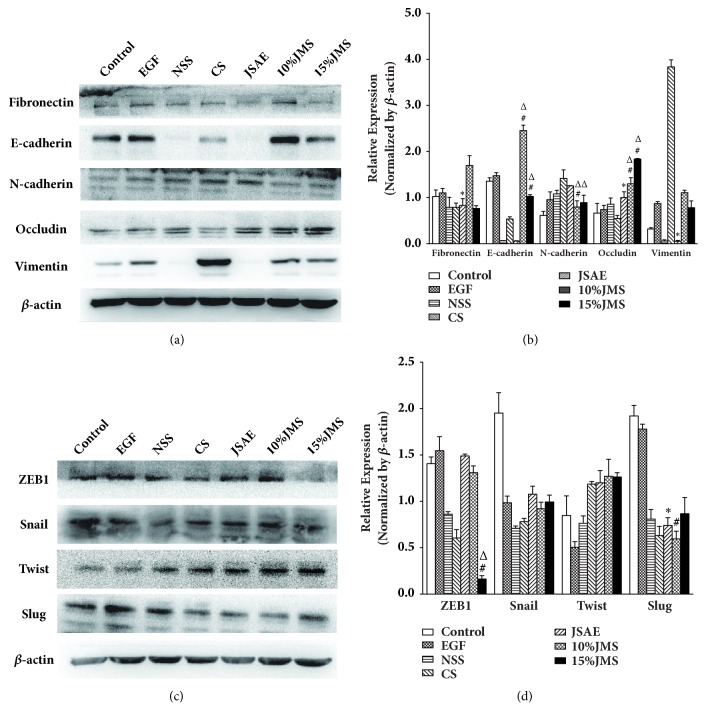
JSD and JSAE reverse EMT by affecting protein levels of EMT regulating factors in SW480 cells after 24 h. The expressions of EMT markers (a) and EMT regulators (c) were analyzed by western blot and quantified by the bar graph (b) (d). ^*∗*^*P* < 0.05, compared with EGF group; ^△^*P* < 0.05, compared with CS group; ^#^*P* < 0.05, compared with NSS group.

The main molecular features of EMT were as following: downregulation of E-cadherin and Occludin, upregulation of N-cadherin and Vimentin [17, 18] as well as ZEB1, Snail, Slug, and Twist that maintained EMT. Western Blot analysis (Figure 6) showed that Occludin was upregulated in JSAE group compared with EGF group, while Vimentin and Fibronectin were downregulated; furthermore, EMT-enhancing Slug was also downregulated. In 10% JMS and 15% JMS groups, negative EMT markers such as E-cadherin and Occludin were upregulated significantly compared with NSS and CS group; conversely, 10% and 15% JMS groups showed a lowered level of N-cadherin (Figure 6(a)). Slug levels decreased in 10% JMS group compared with NSS group, while compared with NSS and CS groups, ZEB1 expression in 15% JMS group was also attenuated (Figure 6(c)). In summary, JSD enhanced negative EMT markers E-cadherin and Occludin, while dampened positive EMT markers such as N-cadherin, Vimentin, Fibronectin, ZEB1, and Slug.

## 4. Discussion

Cancer invasion and metastasis are the major factors that determine the survival of CRC patients. Therefore, an effective control of metastasis is crucial to improve the prognosis of CRC patients. Previous studies have shown that EMT plays an important role in infiltration and metastasis of cancer [10, 19]. EMT is the phenotypic characteristics lost by epithelial cells at the site of tumor infiltration front, coupled with the acquisition of stronger survival, migratory and invasive capacity compared with the cells in the inside area. This is suggested to be the key, triggering step of cancer invasion and metastasis [20, 21]. Tumor cells undergoing EMT acquire features of stem cells with an enhancement of migratory and metastatic capacity [22–24]. Mani et al. and Barrière et al. [25, 26] found that breast cancer cells undergoing EMT possessed stronger metastasis and invasion capacity and resistance to apoptosis and aging. These features were favorable for the spread and survival of tumor cells and the formation of metastatic loci.

E-cadherin is an adhesion molecule which is important in maintaining epithelial phenotype [27]. Partial or complete loss of E-cadherin is closely associated with the high invasiveness, low differentiation and poor prognosis of tumors [28, 29]. E-cadherin downregulation may lead to stronger invasive and metastatic capacity of tumor cells [30–32]. N-cadherin and E-cadherin both belong to cadherin family. The activity of N-cadherin is a function of specific environments [33], and its upregulation is usually found in various tumors [34–38]. EMT is regulated by several transcriptional factors including ZEBs, Snail, and Twist [39–41]. The role of ZEB family is specific to cancer: ZEB1 is important in the invasion and metastasis of colon cancer, while ZEB2 is important in pancreatic cancer, gastric cancer and ovarian cancer [42]. Belonging to the same transcription factor family, Snail and Slug share similar structural properties [43]. Upregulated Twist expression is mainly correlated with poor survival of cancer patients [44–46].

In our current report, through* in vitro* experiments we found that JSD inhibited the migration and invasion of SW480 human colon cancer cells by reversing EMT. Scratch test showed that JSAE/JSD treated cells had the lowest migratory potential and the difference was significant compared with EGF group. These results were further corroborated by transwell migration assays indicating that the number of migratory cells decreased in JMS and JSAE treatment groups. Invasion assays revealed that JSAE/JSD also effectively inhibited invasive potential of SW480 cells. Further mechanistic studies demonstrated EMT inducing factors such as N-Cadherin and Vimentin were highly expressed, while EMT negative regulating factors such as E-cadherin and Occludin being under-expressed in NSS and CS groups. Of note, EMT was enhanced in SW480 cells treated by serum of SD rats, which might contain certain EMT promoting factors. This is also been observed by W. Cui who found that HMGB1 in rats' serum can promote EMT [47]. Nonetheless, JSD upregulated negative EMT markers E-cadherin and Occludin and downregulated positive EMT markers N-cadherin, Vimentin, and Fibronectin as well as ZEB1 and Slug, resulting in reversion of EMT.

## 5. Conclusion

Our results have proven that JSAE inhibited the migration and invasion of SW480 human colon cancer cells by inhibiting EMT. These findings provided the first experimental evidence confirming the efficacy of JSAE in repressing invasion and metastasis of CRC and paving a way for the broader use of JSD in clinic.

## Data Availability

All data generated or analyzed during this study are included in this article.
